# A value chain to improve human, animal and insect health in developing countries

**DOI:** 10.20517/mrr.2023.46

**Published:** 2023-12-08

**Authors:** Gregor Reid

**Affiliations:** ^1^Canadian R&D Centre for Human Microbiome and Probiotics, Lawson Health Research Institute, London N6A 4V2, Ontario, Canada.; ^2^Departments of Microbiology and Immunology, and Surgery, Western University, London N6A 4V2, Ontario, Canada.

**Keywords:** Fermentation, new value chain, arthropods, probiotics, humans, animals, developing countries

## INTRODUCTION

In Mwanza, Tanzania, a group of women produce Fiti yoghurt containing the probiotic *Lactobacillus rhamnosus* GR-1. A single 1-gram sachet with this strain and *Streptococcus thermophilus*, costing $0.50, can produce 100 litres of yoghurt (termed Fiti), millet, or fruit juice^[1-3]^.

This alone represents twenty years of work, an enormous time commitment of Canadian and other countries’ university students, the leadership and dedication of local women and their communities, and consumers who enjoy the products and perceive the health benefits. A value chain has been created involving people of all ages, genders, religions, and color. It includes beehives, purchasing and raising of cows, collection and delivery of milk, millet and fruit, and production, packaging, storage and delivery of end-products. The chain reaches individuals, families, schools, orphanages, and people living on the margins or with health issues not managed by a fragile healthcare system. All from an almost forgotten ancient pastime of fermentation, a process that preserves the food from spoilage and increases nutritional value. The rejuvenated global interest in fermented foods, based on scientific research and clinical evidence, augurs well for these types of initiatives in developing countries^[4]^.

The spin-offs have unexpectedly included the exploration of probiotic bacteria to lower honeybee mortality^[5]^, and the potential to reduce heavy metal and aflatoxin adsorption in children and pregnant women^[6]^. Studies have even been undertaken to help the growth and survival of farmed fish^[7]^, an important commodity in countries surrounding Lake Victoria. The challenge is to sustain these initiatives amidst climate change and food insecurity.

## SUSTAINABILITY

There are numerous factors involved in sustainability, depending on the location. For the initiative in Mwanza, climate change, the environmental setting, the supply chain, and social parameters present important issues.

Climate change has led to extremes of flooding and drought, as well as disease vector migration^[8]^. These affect soil and crops, including pastures for cows. The erosion of fertile ground has implications for the sustainability of produce (milk, fruits, vegetables) required for fermentation. Extremes of heat and cold also affect storage, distribution, and consumer purchasing. In winter, consumers prefer heated products over ones that are served cold, such as yoghurt. This means that to maintain the microenterprise operation, the yoghurt would have to provide benefits when heated or products would need to be developed for room temperature consumption. The heating process that kills the beneficial microbes makes the product a postbiotic (currently defined as “preparation of inanimate microorganisms and/or their components that confers a health benefit on the host”^[9]^). The heating of yoghurt is not generally done in Mwanza, so the alternative of a fermented millet produced and then consumed at room temperature could be useful to sustain the business over the colder weather. While studies have been performed on Fiti^[3]^, none have examined the product as a postbiotic, so the health benefits would need to be examined.

To maximise the benefits of the fermentation process, consideration has been given to spreading the residues of the ferment onto the soil as biofertilizer^[10]^. As microbes and nutrients are integral to soil ecosystems and for sequestering carbon and cycling nutrients, the disruption of their function through heat or flooding has consequences for the food chain^[11]^. In developed countries, mitigation has generally involved the use of chemical fertilizers or genetically engineered plants and microbes. However, apart from the ethical questions this raises, the implementation of these interventions is difficult due to economic and logistical reasons. Biofertilization, however, may be a practical alternative^[12]^ and add to the value chain sustainability.

Another approach to biofertilization could be from algal blooms formed by cyanobacteria on Lake Victoria, upon which Mwanza resides. Harvesting the algae could not only save the fish population, but when placed on soil, these organisms could contribute to eco-friendly fertilization^[13]^. As fishermen live in the communities of Mwanza, teaching them how to harvest the algae could have several positive outcomes and further empower those in the fermented food value chain. Since some of these microenterprises have their own cows, the fertilization of pastures by cyanobacteria would be helpful, in addition to spraying on soil used to grow crops. Furthermore, rather than piling up manure from cows and other livestock, the dung could be spread onto the fields to help with vegetative plant growth and seed germination^[14]^, while fostering the proliferation of earthworms. High in nitrogen, phosphorus, potassium, copper, manganese, and zinc, cow manure contains many lactic acid bacteria. Such application of biofertilizers is feasible in rural African communities, though it will require education, training, and the use of farming equipment in the Mwanza setting, where most microenterprise owners are women without post-secondary education or access to farming machinery such as ploughs and tractors.

A key element of healthy soil is the range of arthropods, which process organic matter translocation, break down and decompose material to release nutrients, regulate water, and maintain the structure of the soil^[15]^. Biofertilizers and animal waste increase organic matter, micronutrients, and soil microbial diversity and can affect soil arthropod groups^[16-18]^. While this makes a rationale for cow manure application in Mwanza, research would be required to identify the microbiota and the response of arthropods to its application. With mosquitos carrying infectious diseases, any increase in their abundance would not be favourable.

An important issue is using locally sourced material rather than relying on importation. This helps with sustainability and affordability for economically challenged residents. This was explored in 2011 by harvesting locally grown *Moringa oleifera* leaves, which contain a range of important nutrients and medicinal properties. The addition of the powder to the probiotic yoghurt was safe and well-received by consumers^[19]^. Given the widespread presence of Moringa trees in East Africa and their ability to grow fast and well in drought, they offer another option for sustainability during climate change. Moringa can also be used to purify water, albeit the process is not simple^[20]^.

The milk supply is not well coordinated in Tanzania, but this has advantages for local Fiti producers as they have easier access to it locally rather than acquiring it from a large collection center. Access to fermentable crops such as millet and fruit diversifies the value chain and increases seasonal business. The ability of the two organisms in the Fiti sachet to produce fermented fruit juice allows for seasonal income and a wider variety of offerings^[2]^. In addition, these organisms ferment almond milk^[21]^ and plant-based foods^[22,23]^.

Concepts such as the microbiome, biofertilizers, and climate change are new to many rural populations in Tanzania, including farmers. Explicit educational material, often using diagrams, is needed to explain these concepts and why they can be important to farming practices. Useful tools include repetitive messaging, field tests to show positive results, and the development of practical steps that can easily be followed. Members of the agricultural community, including highly qualified and experienced personnel, provide a good resource for developing and implementing these programs.

For sustainability, it is important that the broader community is involved. In the past, women were often in the household caring for children and able to produce probiotic fermented foods. However, with the success of the microenterprises and better access to education and training, many women are becoming entrepreneurs and community leaders. This changes the societal dynamics and offers unemployed men an opportunity to further participate all along the value chain by helping to transport the raw materials, produce and package the foods, and then deliver the end-products to retailers, schools, and community locations (meetings, churches, restaurants). The ability of the microenterprises to succeed depends on many other factors including cost of production and packaging, consumer purchasing power, retention of a marketing and sales niche, and flexibility in offering different products.

The health of the broader ecosystem is critical to longevity and expansion. Microbial diversity is a major component in the intestinal tract of humans, animals, and insects. For pollinators whose activities produce much of the food sources, this requires diversity and access to flowering plants. Given the continual loss of habitat, use of toxic chemicals on land (lambda-cyhalothrin, cypermethrin, imidacloprid, glyphosate, and organochlorine^[24]^), and presence of pathogenic bacteria such as *Paenibacillus*, bee colony losses are almost to the point of extreme danger for the global food supply. In Tanzania, challenges to hives also come from wax moths and ants.

African bees are different from North American and European types, being more aggressive and less susceptible to *Varroa* mites. In essence, they tend to be more wild types rather than bred in hives by beekeepers. They produce less honey but are effective pollinators^[25]^. Of those that are used commercially, hives are often hung on greased wires. Their contribution to the value chain is not only in pollination but also in the production of honey, candles, and creams.

To attract and promote wild and commercial bee health, sunflowers could be grown around the pastures and insectary plants such as vegetables, herbs, and flowers could promote beneficial insects over pathogenic ones^[26]^.

Supplementation of hives with probiotics has been successfully tested in North America. A three-strain probiotic counters *Paenibacillus larvae* (deadly bacterial pathogen) and *Varroa destructor* (ectoparasitic mite)^[27,28]^. Whether these strains would be effective in Tanzania requires research, as does the potential to spray probiotic strains into the flower pollen as a means to deliver them to the bees.

## EXPANSION

The number of microenterprises in the Fiti industry in the Mwanza region has grown to around 80 over the past twenty years, and along with others across the country, they cater to over 100,000 consumers^[3]^. In order to retain these units and expand across the region and country, several things are worth exploring in addition to education, product expansion, and the improvement of soil discussed above.

The well-being of cattle could be improved in two ways: firstly, by granting them access to pastures abundant in grass, and secondly, by providing them with fermented foods. Corn and barley silage and alfalfa haylage are common fermented forages for cows in North America with benefits to manure spreading and soil health, perennial growth, pollinator proliferation, and carbon sequestration^[29]^. The fermentation process increases nutrients, improves digestion, and can result in higher milk yields depending on the composition of the silage^[30]^. Consumption of fermented feed also improves the reproductive performance and overall health of the cows. Since the microenterprises are already fermenting milk, vegetables, grains, and fruit for human use, the addition of animal-targeted ferments would expand the business and foster sustainability.

Another means of expansion is through the management of water. In the rainy season, the water is not collected on farms but tends to simply flow along paths, roadways, and eventually into drainage systems. By pooling it in a retention pond, the farmers could utilize it to irrigate crops in the dry season. To avoid it becoming a source of mosquito replication, dried pellets of *Bacillus thuringiensis* could be added to the water, killing the larvae^[31]^.

Households could also retain water from rooftops, a practice used in Bermuda because of the limestone available there. Without piped water, adults and children frequently have to travel to wells to obtain water for household and microenterprise use, thereby having less time to produce fermented food, the revenue from which helps with schooling and healthcare.

With women-led networks providing advice and assistance with book-keeping, positioning and marketing of products and helping to solve production issues, a system that links the microenterprises via cell phones is being explored. This enables shared experiences and collaboration.

Another opportunity to expand local microenterprises is through edible insects, such as the grasshopper *Ruspolia differens*. A study showed high demand for edible arthropods in the Lake Victoria basin^[32]^. These can be blanched, boiled, toasted, and deep-fried, providing a range of protein, fibre, α-linolenic acid, flavonoids, iron, and zinc^[33]^. Importantly, safe storage and propagation are vital to avoid the release and swarming of these plant-devouring insects. Already, mayflies of the genus *Povilla* (Polymitarcyidae) are dried and used in meal preparation around Lake Victoria^[34]^. One potential problem is that aquatic insects can bioaccumulate toxic heavy metals^[35]^ and pathogens from raw sewage present in the lake. A range of other arthropods can be found in the lake and used as a food source, but the Fiti fermented food production units have so far not considered expanding to fish or arthropods. This could change through the expansion of the value chain since fermentation of fish has long been a tradition in Africa^[36]^. If a core facility could be built in Mwanza to not only produce and distribute more products but also isolate, grow, dry, and package sachets containing various microbial fermenters, there would be a means to not only improve the health and well-being of the population, but also better utilize the food sources at hand.

## APPLICATIONS TO DEVELOPED COUNTRIES

World Vision recently reported that an estimated 719 million people are living in poverty (less than $2.15 per day), while 1.2 billion people in 111 developing countries live in multidimensional poverty (https://www.worldvision.org/sponsorship-news-stories/global-poverty-facts). As inequality grows and economic expansion becomes environmentally unsustainable, questions must be asked about how populations access food resources and the nutritional value of foods they can afford. Poverty and purchasing cheap, poorly nutritious food can lead to obesity and malnourishment^[37]^. Furthermore, a case has been made that the current food production system is adversely affecting planetary and human health^[38]^.

In Canada, there is a supply management system under the national agricultural policy that controls the supply of dairy, poultry and eggs. This is designed to offer stability for producers and processors, but a consequence is that purchasers are at the mercy of prices set by the conglomerate. There are numerous examples of global food systems that lead to affordable but poor-quality foods being purchased by people in poverty. The question is, could the microenterprise system based on fermentation established in Tanzania work in Canada?

The answer is not simple. Access to raw milk is difficult because of the supply management rules. Production of food for sale requires rigorous Health Canada assessment for safety, albeit churches and local organizations provide free meals to needy people without government approvals. Setting up a business and managing its finances is laborious. Nevertheless, in practical terms, small groups of people could produce products like Fiti under a microenterprise focusing on consumers below the poverty line. In doing so, unemployed youth and adults could become engaged in the value chain. One such initiative to sell Fiti yoghurt is being explored in London, Ontario, specifically at a Youth Opportunities Unlimited cafeteria located in an area of the city frequented by unemployed, homeless, and drug-addicted people.

**Figure 1:**
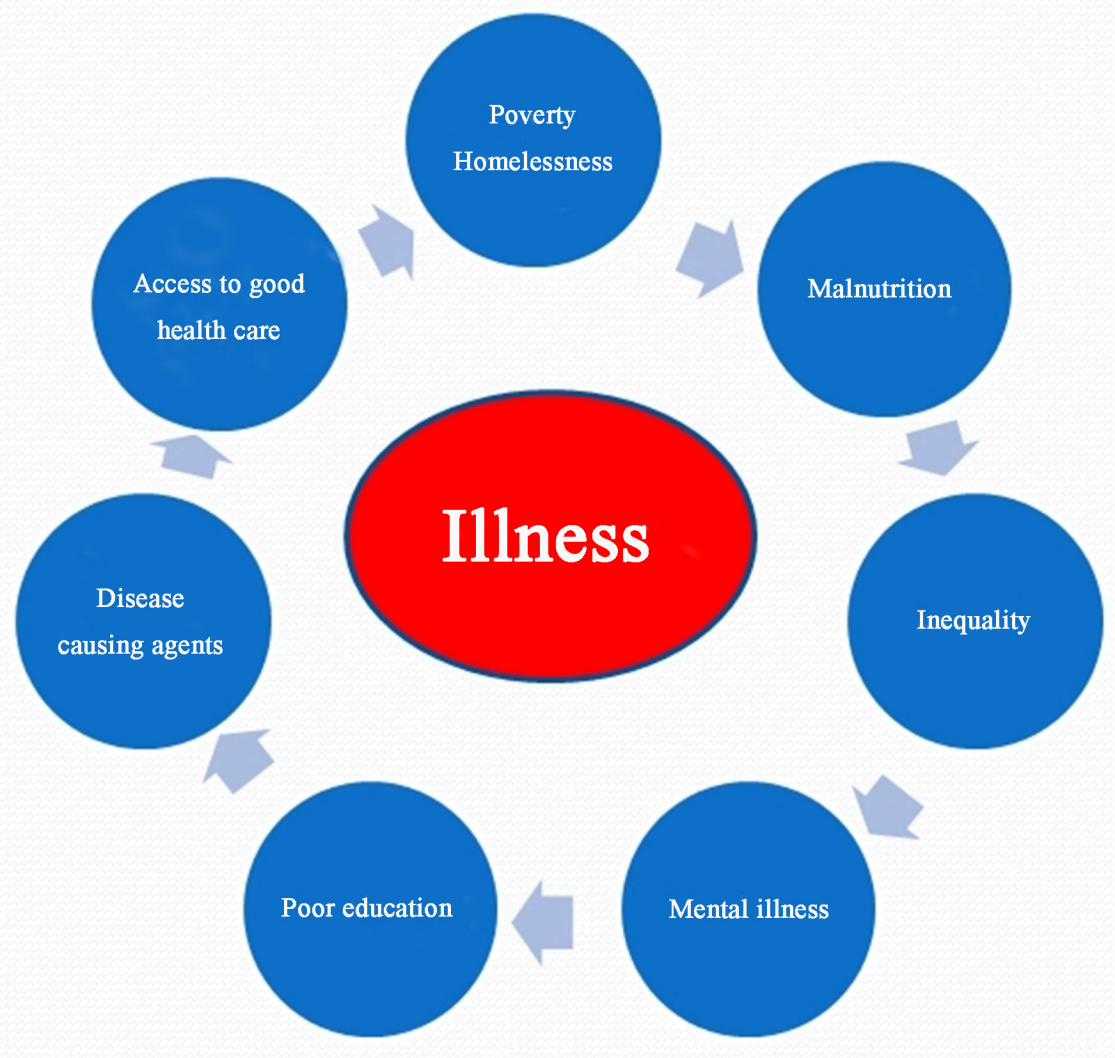
Factors that influence illness and recovery.

Another application could be through a microenterprise providing hospitals with fermented food to aid patient healing. Given the safety record of products like Fiti in HIV patients^[39]^, probiotics and prebiotics for trauma and surgery patients^[40]^, and fermented foods for various health issues^[41]^, it is surely a concept worthy of consideration. After all, invariably, illness does not have a single cause and cure [Figure 1].

## IN SUMMARY

The rapidly growing realization that beneficial microbes are important for global health and human survival has offered endless possibilities for their application. This cannot only benefit people in rich countries. An initiative that started in Tanzania to teach women primarily how to produce probiotic fermented yoghurt has blossomed in the East African region and beyond. The new food value chain that it has created empowers all genders and ages and is now expanding to encompass different food sources, including grains, vegetables, and fruit. The potential to further expand the chain through microbes that promote pollinator health, encourage beneficial arthropods, and reduce heavy metal contamination from fish has great potential, not only for Tanzania but for many developing and developed countries. While the trend of mass starvation is declining, malnutrition is still widespread, and the growing population requires new food sources. In Argentina, the use of probiotic yoghurt nationwide for school children is an excellent example of how a grassroots initiative can benefit a country’s population^[42]^. The use of beneficial microbes can be the cornerstone of One Health, integrating microbes with systems that promote health for people, animals, arthropods, and the wider ecosystem.

## DECLARATIONS

### Copyright

© The Author(s) 2023.
